# High Affinity Antigen Recognition of the Dual Specific Variants of Herceptin Is Entropy-Driven in Spite of Structural Plasticity

**DOI:** 10.1371/journal.pone.0017887

**Published:** 2011-04-22

**Authors:** Jenny Bostrom, Lauric Haber, Patrick Koenig, Robert F. Kelley, Germaine Fuh

**Affiliations:** 1 Department of Antibody Engineering, Genentech Inc., South San Francisco, California, United States of America; 2 Department of Protein Engineering, Genentech, Inc., South San Francisco, California, United States of America; The Scripps Research Institute, United States of America

## Abstract

The antigen-binding site of Herceptin, an anti-human Epidermal Growth Factor Receptor 2 (HER2) antibody, was engineered to add a second specificity toward Vascular Endothelial Growth Factor (VEGF) to create a high affinity two-in-one antibody bH1. Crystal structures of bH1 in complex with either antigen showed that, in comparison to Herceptin, this antibody exhibited greater conformational variability, also called “structural plasticity”. Here, we analyzed the biophysical and thermodynamic properties of the dual specific variants of Herceptin to understand how a single antibody binds two unrelated protein antigens. We showed that while bH1 and the affinity-improved bH1-44, in particular, maintained many properties of Herceptin including binding affinity, kinetics and the use of residues for antigen recognition, they differed in the binding thermodynamics. The interactions of bH1 and its variants with both antigens were characterized by large favorable entropy changes whereas the Herceptin/HER2 interaction involved a large favorable enthalpy change. By dissecting the total entropy change and the energy barrier for dual interaction, we determined that the significant structural plasticity of the bH1 antibodies demanded by the dual specificity did not translate into the expected increase of entropic penalty relative to Herceptin. Clearly, dual antigen recognition of the Herceptin variants involves divergent antibody conformations of nearly equivalent energetic states. Hence, increasing the structural plasticity of an antigen-binding site without increasing the entropic cost may play a role for antibodies to evolve multi-specificity. Our report represents the first comprehensive biophysical analysis of a high affinity dual specific antibody binding two unrelated protein antigens, furthering our understanding of the thermodynamics that drive the vast antigen recognition capacity of the antibody repertoire.

## Introduction

Monoclonal antibodies are typically specific toward single antigens. The exquisite specificity of antibody-antigen interactions is one main reason behind the success of antibodies as targeted therapeutics [1]. However, there is increasing appreciation for the prevalence of polyreactive or multispecific antibodies and their potential roles in the immune recognition function of the antibody repertoire [2], [3], [4], [5]. Polyreactivity is in fact a common phenomenon among the precursors of antibodies on pre or pro B lymphocytes and is often associated with self reactivity. Although the receptor editing process eliminates many of these self and polyreactive clones [6], [7], mature B cells that secrete polyreactive antibodies in the periphery have been reported [2], [5], [8]. The finding that antibodies may become polyreactive through somatic mutations further implicates the prevalence of polyreactive antibodies in the antibody repertoire [9], [10]. The ability of single antibodies to interact with more than one antigen has been proposed to expand the effective size of the antibody repertoire, which theoretically is restricted by the limited number of B lymphocytes, each expressing only one antibody [4], [9], [11], [12]. Moreover, there is interest in antibodies that may target more than one antigen molecule for therapeutic applications.

Many have endeavored to understand the molecular mechanisms of antibody polyreactivity or multi-specificity. Antibodies have been shown to bind distinct antigens that are conserved chemically and/or structurally with high affinity [13], [14], [15], [16], [17], [18], [19], [20]. However, a single antibody able to interact with more than one antigen epitope devoid of homology may have greater impact on the antigen recognition capacity of the immune repertoire. The multi-specific interactions of antibodies have been studied primarily by first identifying the additional “antigens” toward the antibody of interest from combinatorial libraries of peptides, small molecules or proteins [4], [12], [13]. Some antibodies were shown to rely on conformational variability, or structural plasticity of the antigen-binding site to adapt to distinct antigens [4]. Other antibodies were shown to utilize different regions of the antigen-binding site to engage various antigens without involving significant structural plasticity [12], [13], [19], [21]. However, since these polyreactive antibodies derived their secondary antigens from repertoire selection, the identity of the secondary antigen cannot be pre-determined. Further, the binding affinities for the selected secondary antigens are often quite weak. Insights into the mechanism of high affinity, multi-specific antibody interactions are still lacking.

We recently described a strategy to generate dual specific antibodies, or two-in-one antibodies, toward two defined antigens *de novo* by recruiting a second specificity to the antigen binding site of a monospecific antibody [22]. We demonstrated the strategy by first constructing a combinatorial phage-displayed library randomizing the light chain (LC) complementarity determining regions (CDRs) of Herceptin® (trastuzumab), a humanized antibody originally derived from mouse hybridoma. From this library, we isolated many variants that can bind a secondary antigen while maintaining binding towards their primary antigen HER2. We believe the strategy can be applied to evolve the specificity of other antibodies because, like Herceptin, many natural antibodies utilize the heavy chain (HC) CDRs as the main antigen binding determinants, and therefore can tolerate some mutation in the LC CDRs. The sequences of the derived dual specific antibodies demonstrate that a variety of limited mutations are sufficient for antibodies to recruit a second specificity. The dual specific antibody bH1 and its affinity-improved variants represent the first such antibodies that bind tightly to two unrelated protein antigens, HER2, the primary antigen, and VEGF, the secondary antigen (**Table 1**) [22].

**Table 1:** CDR Sequence alignment of Herceptin and bH1 variants.

	Light Chain																	Heavy Chain																			
	28	29	30	30a	30b	30c	30d	31	32	50	51	52	53	91	92	93	94	30	31	32	33	50	51	52	52a	53	54	55	56	57	58	95	96	97	98	99	100
Herceptin	D	V	N	.	.	.	.	T	A	S	A	S	F	H	Y	T	T	K	D	T	Y	R	I	Y	P	T	N	G	Y	T	R	W	G	G	D	G	F
bH1		I	P	R	S	I	S	G	Y	W	G		Y																								
bH1-81	N	I	A	K	T	I	S	G	Y	W	G					S	S																				
bH1-44	N	I	A	K	T	I	S	G	Y	W	G					S	S	S	G							S	E						V		V		

Information tabulated based on [22].

Light chain and heavy chain CDR residues of bH1 and variants that differ from Herceptin are shown.

**Figure 1:**
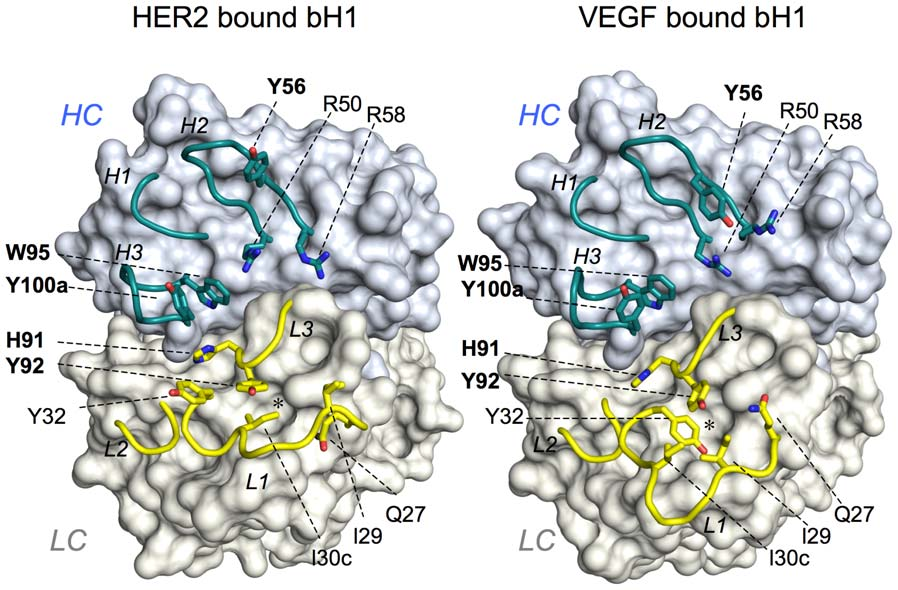
The distinct CDR conformations of bH1. The three HC CDRs (cyan) and three LC CDRs (yellow) of bH1 in complex with HER2 (PDB code 3BE1) or VEGF (PDB code 3BDY) are shown in cartoon representation with the rest of bH1 structure as surface. Selected residues are shown in stick representation: LC-I29, Y32 and I30c are specifically important for bH1-44/VEGF binding and HC-R50 and R58 are specifically important for bH1-44/HER2 binding whereas LC-H91 and Y92, and HC-Y56, W95 and Y100a (in bold) are residues important for the bH1-44 interaction with both antigens as well as the Herceptin interaction with HER2. Note the highly distinct conformations of CDR-L1, the adjustment of the side chains of highlighted residues, and the side chains of LC-I30c and LC-Y32 that alternatively occupy the nearby cavity (*) in HER2 bound bH1 or VEGF bound bH1, respectively. See Figure S1 movie (Movie S1) morphing the two structures, which highlights the extent of the conformational adjustment for dual interaction.

The crystal structures of bH1 in complex with HER2 and VEGF reveal that bH1 makes contact with its two antigens via a similar set of surface residues [22]. However, the surface contour of bH1 buried in either complex is compatible only with the antigen in complex and molecular acrobatics is clearly involved for the antibody to bind its two antigens (**Figure 1**, **Figure S1**). In contrast, Herceptin Fab exhibits minimal structural variability based on the available structures of free and HER2-bound Herceptin Fab [23], [24] (**Figure S2**).

To understand the dual antigen recognition of these antibodies evolved from a monospecific antibody and the effect of significant structural plasticity on binding energetics, here we analyzed the biophysical and thermodynamic properties of the antigen interaction of bH1 and its affinity-improved variants including bH1-44 and compared to those of Herceptin. While bH1 and bH1-44 share many characteristics with their parent antibody Herceptin (binding epitopes, affinity, kinetics, and critical CDR residues), the thermodynamic profile of the dual specific variants deviate from that of Herceptin. Notably, the bH1 interactions with HER2 and VEGF were characterized by large favorable entropy changes whereas the Herceptin interaction was enthalpically driven. We dissected the total entropy changes to examine the contribution of the hydrophobic effect (desolvation entropy) to the antigen interaction and the penalty from reduction of conformational freedom (conformational entropy) upon antigen binding. We also examined the accessibility of bH1-44 for binding its two antigens compared to that of Herceptin for HER2. We determined that the increased structural plasticity of bH1 antibodies relative to Herceptin appeared not to negatively impact the binding free energy. Central to the high dual affinity of the bH1 antibodies is the structural plasticity, with low energetic cost, in addition to the hydrophobic desolvation energy that drives the interactions.

## Results

### The affinity-improved HER2/VEGF dual-specific antibody retains similar binding affinity and kinetics as Herceptin

**Figure 2:**
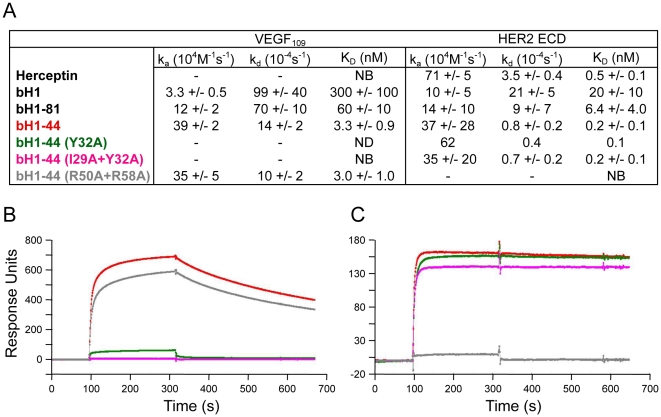
Antigen-binding affinity and kinetic of the bH1 variants and Herceptin. (A) k_on_ and k_off_ and dissociation constant (K_D_) of Fabs binding to immobilized VEGF or HER2 are determined by SPR measurements (See Methods). The errors represent the standard deviations based on at least three independent experiments. bH1-81 was measured only once. Hence no error estimation is available. NB = No binding detected. ND = Not determined as the interaction was too weak to assess. The representative binding responses over time of 0.5 µM Fab bH1-44 (red), bH1-44 (LC-Y32) (green), bH1-44(LC-I29A+Y32A) (magenta) and bH1-44(HC-R50A+R58A) (gray) to immobilized VEGF_109_ (B) or HER2-ECD (C).

We determined the binding kinetics for variants of the dual-specific antibody bH1 and Herceptin as Fabs to immobilized VEGF (the receptor binding domain, VEGF_8–109_) or HER2 (the extracellular domain, ECD, of HER2) using surface plasmon resonance (SPR) measurements (**Figure 2**). The bH1 Fab/VEGF interaction is characterized by a moderate rate of association (k_on_ = 4×10^4^ M^−1^ s^−1^) and a fast rate of dissociation (k_off_ = 0.01 s^−1^) at 30°C, which results in a dissociation constant (K_D_) of 300 nM. The bH1/HER2 interaction (K_D_ = 20 nM, k_on_ = 1×10^5^ M^−1^ s^−1^, k_off_ = 2×10^−3^ s^−1^) is 40-fold weaker than the Herceptin Fab/HER2 interaction (K_D_ = 0.5 nM, k_on_ = 7×10^5^ M^−1^ s^−1^, k_off_ = 4×10^−4^ s^−1^ at 30°C). Using two consecutive rounds of affinity improvements, bH1 was improved first to bH1-81 then to bH1-44 with incremental improvements in both on-rates and off-rates for VEGF and HER2 interactions compared to the parent bH1 (**Figure 2**). As a result, bH1-44 binds HER2 with similar affinity and kinetics as Herceptin.

### Many interactions between Herceptin and HER2 are retained in the dual interaction

**Figure 3:**
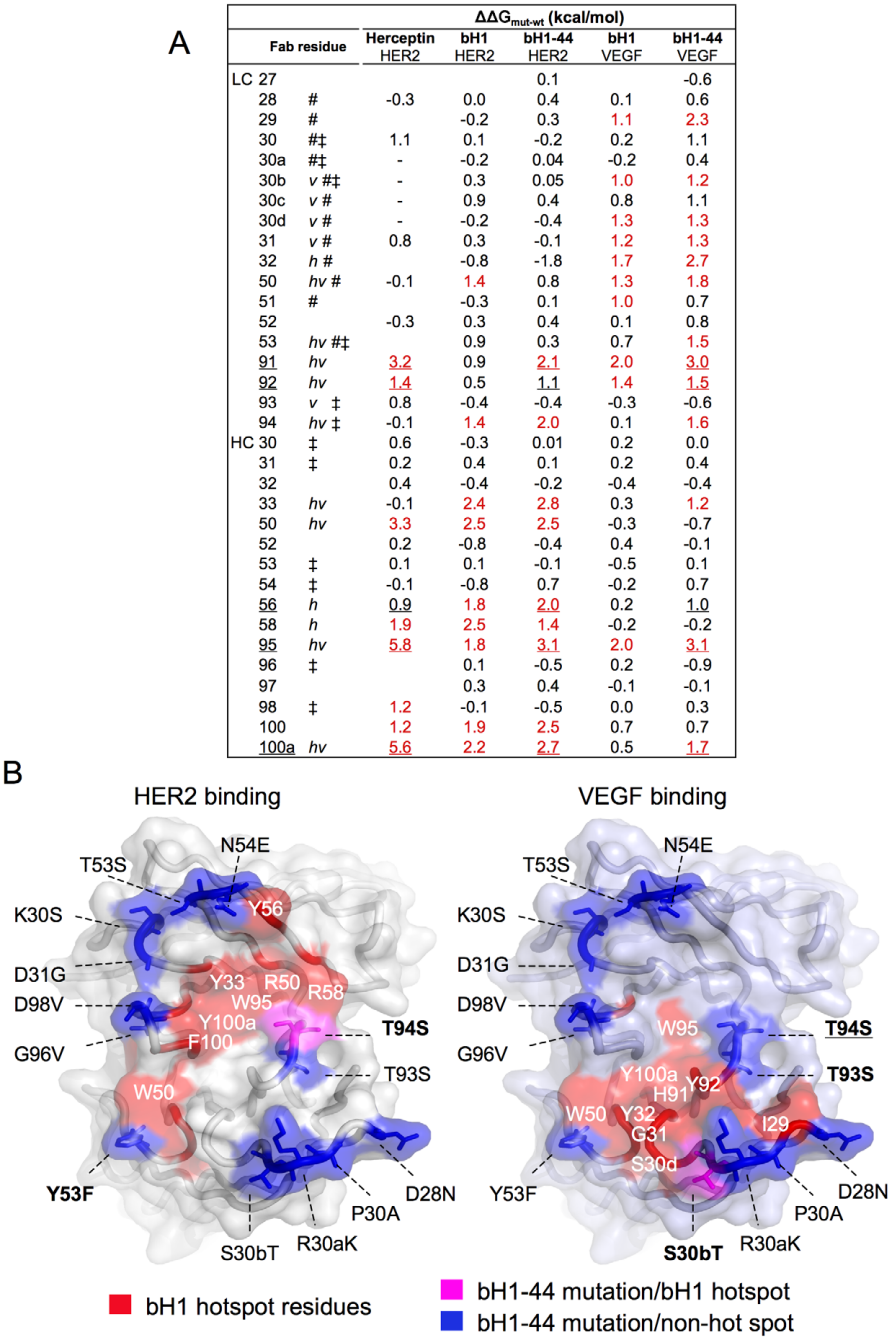
Mapping of the evolved dual specific interactions. The changes in binding free energy (ΔΔG_mut-wt_) when a Fab residue is mutated to alanine (based on data from Kelley et. al. Biochemistry, 1993, Bostrom et. al., Science, 2009, and the current study) are shown (A). Amino acids are numbered as in Table 1 and denoted with *h* or *v* if making structural contact with HER2 or VEGF (within 4.5 Å), respectively, # if mutated from Herceptin to bH1, and ‡ if mutated from bH1 to bH1-44. Underlined residues are energetically important (ΔΔG_mut-wt_ greater than ∼1 kcal/mol, also underlined) for Herceptin/HER2, bH1-44/HER2 and bH1-44/VEGF interactions indicating importance for all three interactions. Hotspot residues highlighted in red are those with ΔΔG_mut-wt_ greater than ∼10% of the total binding free energy of each interaction. “-” denotes no residue in Herceptin at the position. Values not determined are left blank. (B) bH1-44 mutations (from bH1) are mapped to locate predominantly outside of functional hotspots and structural contact for HER2 binding (left) or VEGF binding (right) on the surface of the bH1 paratope modeled with side chains of bH-44. The residues are colored red if they are bH1 hotspot residues for HER2 or VEGF binding, blue if the residues are mutated from bH1 to bH1-44 and not part of bH1 hotspot for each respective interaction, or magenta if the mutated residues are part of bH1 hotspot. A mutation is bolded if the residue is a structural contact site in the respective bH1 interface or underlined if the mutated residue gains significant functional importance.

Alanine-scanning mutagenesis studies of bH1 and bH1-44 [22] and Herceptin [25] identified the CDR residues that significantly contribute to the binding free energy (ΔG). Hotspot residues are referred to sites where alanine substitution results in a reduction of 10% or greater of the total ΔG. Analysis of the mutagenesis mapping shows that bH1 and bH1-44 have conserved all but one of the Herceptin/HER2 hotspot residues in their sequences. Further, the majority of Herceptin's hotspot residues remain important for both bH1 and bH1-44 to bind HER2 (**Figure 3A**) (**Table 1**).

The VEGF binding hotspot contains a different set of residues when compared to the HER2 hotspot, however, there are five mostly centrally located CDR residues that contribute significantly to all three high-affinity Herceptin/HER2, bH1-44/HER2 and bH1-44/VEGF interactions. This includes two residues in the light chain (LC; His91 and Tyr92) and three in the heavy chain (HC; Tyr56, Trp95 and Tyr100a). While the main chain of these residues are at a similar position in both bH1 structures as in the Herceptin structures, their side chains appear to undergo various extents of adjustments in order to optimize interaction with the two antigens (**Figure 1**, **Figure S1**). In particular, Tyr56 of CDR-H2 exhibits a significant difference in conformation. The rearrangement of this residue is important since the unique side chain conformation in the VEGF-bound bH1 is not compatible for both bH1 and Herceptin to bind HER2. Therefore, the VEGF binding ability of bH1 appears to co-opt some of the HER2 binding residues of Herceptin, albeit with conformational adjustments.

### bH1-44 largely conserves the hotspot residues of bH1

Nine out of ten VEGF hotspot residues and eight out of nine HER2 hotspot residues of bH1 are also hotspot residues for bH1-44 (**Figure 3**). Further, most of the mutations that distinguish bH1 from bH1-44 involve conservative changes in amino acid residues located at the periphery of the bH1 hotspots that are not in direct contact with either antigen based on the bH1 complex structures (**Figure 3B**). The conservation of both hotspot and periphery residues suggests that the molecular structure of the bH1-44 complexes should be similar to that of bH1, with the affinity-improving mutations likely playing indirect or allosteric roles. Moreover, residues of CDR-L1, which exhibits highly distinct conformations in the two bH1-complexes, are similarly important for both antibodies. The severe effect of mutating Tyr32 of CDR-L1 in bH1 and bH1-44 to either alanine or phenylalanine on VEGF binding is striking and demonstrates the importance of CDR-L1 conformation for both bH1 and bH1-44 to bind VEGF (**Figure 2**, **Figure S3**) [22]. Based on the mutagenesis mapping of bH1 and bH1-44, we generated Ile29A+Tyr32A (LC) or Arg50A+Arg58A (HC) mutations in bH1-44, which completely disrupted the binding to VEGF or HER2, respectively while maintaining the binding affinity and kinetics for the other antigen (**Figure 2**). We also confirmed that the Arg50A+Arg58A (HC) mutations disrupted Herceptin's binding to HER2 completely (**Figure S4**), which corroborates that bH1-44, much like bH1, maintains the HER2 interaction of Herceptin. Hence, the approximately 100-fold improvement of dual affinity (from 20 nM to 0.2 nM in K_D_ for HER2, from 300 nM to 2 nM in K_D_ for VEGF) is an optimization of the existing interactions of bH1.

### Favorable entropy change is the hallmark of the dual interaction of bH1 and bH1-44

To determine the thermodynamic profiles of the high-affinity dual interaction, we first assessed the overall thermal stability of the dual specific Fabs by performing thermal denaturation experiments using differential scanning calorimetry (DSC) (**Figure S5**). The melting temperatures (T_M_) of the dual specific variants as Fabs (77°C, 76°C, 74°C for bH1, bH1-81 and bH1-44, respectively) are slightly lower than that of Hercepin Fab (83°C) [26], but they are within the range of other therapeutic antibodies [27]. These Fabs are sufficiently stable for the biophysical studies described below. Further, the two antigen constructs, VEGF_8–109_ and HER2-ECD, have been shown previously to be suitable for thermodynamic studies [26], [28].

We then determined the enthalpic (ΔH) contribution to the interaction between the bH1 Fab variants and VEGF or HER2 using isothermal titration calorimetry (ITC). The antigen binding affinities of bH1-44 or Herceptin Fabs are too high for accurate determination by ITC; therefore, we utilized SPR measurements to determine the dissociation constants (K_D_). Changes in free energy (ΔG) and entropy (ΔS) upon binding were calculated from the K_D_ and ΔH as described in Methods.

**Figure 4:**
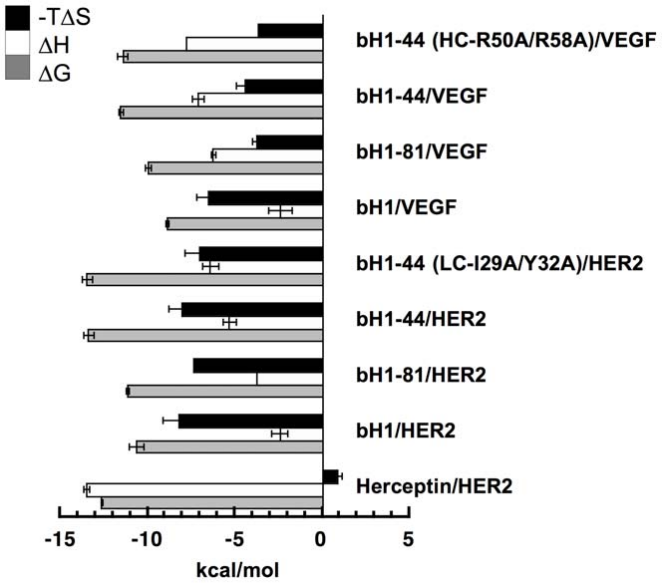
Thermodynamic profiles of the bH1 variants and Herceptin. The entropic component (−TΔS) and enthalpic component (ΔH) of the binding free energy (ΔG) measured at 30°C in phosphate buffer (pH 7.4) are shown in kcal/mol. ΔG was derived from the dissociation constant (K_D_) measured with SPR (See Figure 2) (ΔG = RT*ln*K_D_), and ΔH was measured using ITC. −TΔS was calculated from the ΔG and ΔH according to −TΔS = ΔG−ΔH. Error bars of ΔG and ΔH represent standard deviation of three independent measurements (exception: the enthalpies of the bH1-81/HER2 and bH1-44(R50A/R58A)/VEGF interactions were measured only once, thus no error bar), and the errors of −TΔS are combined errors of ΔG and ΔH.

Interestingly, the interactions of bH1 with VEGF and HER2 display markedly similar thermodynamic properties (**Figure 4**, **Figure S6**). Both interactions are exothermic (ΔH = −2.4 kcal/mol as measured at 30°C in phosphate buffer at pH 7.4) and are predominantly driven by a highly favorable entropy change (−TΔS = −6.5 and −8.2 kcal/mol for VEGF and HER2, respectively). In contrast, Herceptin Fab interaction with HER2 appears to be primarily driven by favorable enthalpy changes (ΔH = −13.5, −TΔS = 0.9 kcal/mol) as determined side-by-side with bH1 antibodies in this study, which is similar to the values described previously [26].

The high-affinity interactions of bH1-81 and bH1-44 with antigens are also characterized by favorable enthalpy and entropy (**Figure 4**). For both VEGF and HER2 interactions, the affinity improvement is associated with a more favorable enthalpy change: ΔH = −7.1 (bH1-44) versus −2.4 kcal/mol (bH1) for VEGF; ΔH = −5.3 (bH1-44) versus −2.4 kcal/mol (bH1) for HER2 at 30°C. Further, the two double mutants of bH1-44 that retain high affinity for either HER2 (LC-I29A/Y32A) or VEGF (HC-R50A/R58A) exhibit the same profile of favorable enthalpy and entropy contributions as bH1-44 (**Figure 4**). Hence, the contrasting binding thermodynamics of bH1 antibodies compared to Herceptin is consistently demonstrated in both binding functions of many bH1 variants.

To further confirm the favorable entropy changes associated with the interactions of the bH1 antibodies, we determined the contribution of protonation effects to the observed ΔH values. For protein-protein interactions, the measured ΔH can sometimes include heat effects related to protonation/deprotonation of the protein and buffer components. This can lead to inaccuracy in the calculation of ΔS for binding. We used the commonly accepted procedure [29] to examine and correct for these effects. We measured the enthalpy changes of the bH1-44 and Herceptin interactions in Tris buffer (pH 7.5) (ΔH_Tris_), which has a large ionization enthalpy (ΔH_TrisBuffer_ = 11.1 kcal/mol, 30°C) [30] and compared these to the values measured in phosphate buffer (pH 7.4)(ΔH_PBS_), which has minimal ionization enthalpy [31]. For bH1-44 binding to VEGF, no significant difference in ΔH was observed in the two buffers, indicating that no significant exchange of protons occurs between the proteins and the buffer in this interaction (**Table 2**). For the Herceptin/HER2 and bH1-44/HER2 interactions, however, significant differences were observed indicating that protonation of the proteins occurs in association with HER2 binding. The change in enthalpy corresponds to approximately 0.5 protons ((ΔH_Tris_−ΔH_PBS_)/ΔH_TrisBuffer_ = −8.0−(−13.5)/11.1 = 0.5) for each Herceptin/HER2 interaction and 0.2 protons (−3.1−(−5.3)/11.1 = 0.2) for each bH1-44/HER2 binding event. Given the pH of the solutions and the pKa (6.0) of histidine, the most likely scenario is protonation of one histidine side chain for every two or five binding events, respectively. Assuming that the enthalpy for protonation of the histidine is similar as for imidazole (−8.7 kcal/mol, 30°C) [30], the enthalpy change upon binding was corrected by 0.5×(−8.7) = −4.35 kcal/mol for Herceptin and 0.2×(−8.7) = −1.74 kcal/mol for bH1-44. With the correction, the Herceptin/HER2 interaction is still highly exothermic ΔH^corr^ = −9.2 kcal/mol ( = −13.5−(−4.35)) and associated with a small favorable entropy contribution (−TΔS^corr^ = −3.4 kcal/mol, 30°C), whereas the bH1-44 interaction with HER2 is indeed characterized by a small enthalpy contribution (ΔH^corr^ = −3.6 kcal/mol ( = −5.3−(−1.74)) augmented by a large entropy contribution (−TΔS^corr^ = −9.8 kcal/mol, 30°C ) (**Table 2**).

**Table 2:** Thermodynamic parameters of Fab interactions with VEGF and HER2.

	ΔH_PBS_	ΔH_Tris_	ΔH^CORR^	ΔS_TOT_	ΔCp	ΔS_CONF_	ΔS_SOLV_	ΔS_RT_
	kcal mol^−1^	kcal mol^−1^	kcal mol^−1^	calK^−1^ mol^−1^	calK^−1^ mol^−1^	calK^−1^ mol^−1^	calK^−1^ mol^−1^	calK^−1^ mol^−1^
**bH1-44/VEGF**	−7.1	−8.0	−7.1	16	−400	−72	96	−8.0
**bH1-44/HER2**	−5.3	−3.1	−3.6	32	−440	−65	105	−8.0
**Herceptin/HER2**	−13.5	−8.0	−9.2	11	−370	−70	89	−8.0

ΔΗ_PBS_ and ΔΗ_Tris_ were determined by ITC in PBS (pH 7.4) or Tris (pH 7.5) buffer at 30°C.

ΔΗ^CORR^, corrected ΔΗ, was calculated as described in Results.

ΔS_TOT_, net total ΔS, was calculated from ΔΗ^CORR^ and ΔG as described in Methods.

ΔCp, heat capacity change, was calculated by linear regression of the temperature versus ΔH (Figure S6 and Methods). ΔCp of Herceptin/HER2 (−370 calK^−1^ mol^−1^+/−30 calK^−1^ mol^−1^) is based on [26]. Standard errors of ΔCp measurements for bH1-44/VEGF and bH1-44/HER2 interactions are 7 and 13 calK^−1^ mol^−1^, respectively.

ΔS_SOLV_ = ΔCp*ln*(T/Ts*), where T = 303.15 K and Ts* = 385.15 K.

ΔS_RT_ was estimated to −8 cal K^−1^ mol^−1^ for a protein binding reaction.

ΔS_CONF_ was calculated as ΔS_CONF_ = ΔS_TOT_−ΔS_SOLV_−ΔS_RT_, see Methods.

Standard errors of the ITC measurements were generally within 10% (See Figure 4).

### Hydrophobic interactions drive the binding of bH1 variants and Herceptin

Favorable entropy changes in protein-protein interactions commonly arise from desolvation, *i.e.* the hydrophobic effect. Expulsion of ordered water from the apolar surface upon ligand binding increases the total entropy of the system. To investigate the importance of desolvation, we determined the heat capacity change (ΔCp) from the temperature dependence of ΔH for three interactions: bH1-44 Fab with VEGF or HER2 and Herceptin Fab with HER2 (**Table 2**, **Figure S7**). Highly negative ΔCp values were measured for bH1-44/HER2 and bH1-44/VEGF interactions (**Table 2**) indicating an important contribution to binding from the hydrophobic effect (Kauzmann, 1959). The magnitudes of the ΔCp values are consistent with the approximately 60% hydrophobic composition of the interfaces (**Table 3**), which is in range of what has been observed in typical protein-protein or antibody-antigen interfaces. For the Herceptin/HER2 interaction (also ∼60% apolar), the previously determined ΔCp value of −370 cal/molK^−1^ [26] is slightly smaller than the ΔCp of bH1-44/HER2 (−440 cal/molK^−1^), but still indicates the important role of the hydrophobic interaction in Herceptin/HER2 binding. The greater hydrophobic effect in bH1-44 compared to Herceptin is consistent with a larger buried apolar surface estimated in the interface of bH1/HER2 complex compared to Herceptin/HER2 complex (988 versus 910 Å^2^, respectively, **Table 3**).

**Table 3:** The area and property of the interfaces in the Fab/antigen complexes.

Interface	bH1/VEGF				bH1/HER2				Hercepin/HER2			
	bH1	VEGF	Combined	Fraction	bH1	HER2	Combined	Fraction	Herceptin	HER2	Combined	Fraction
**Polar**	311	295	606	40%	308	282	591	37%	307	308	614	40%
**Apolar**	438	462	900	60%	470	518	988	63%	441	469	910	60%
**Total**	749	757	1506		779	800	1579		747	777	1524	

All numbers in Å^2^ unless otherwise indicated.

The polar and apolar area of the interfaces was assessed using XSAE (See Methods). The area was calculated using a probe with a radius of 1.4 Å.

Based on the observed ΔCp, we next calculated the theoretical apolar surface area buried in the interface (ΔA_np_) as previously described [26], [32]. Large deviations of the calculated buried ΔA_np_ from the observed buried ΔA_np_ in the structure of complexes indicate significant effect of local folding upon binding, e.g. induced-fit. Examples in the literature include the interaction of a T cell receptor (TCR) with an antigen peptide-MHC complex (pMHC) [33] and the TATA binding protein interaction with the adenovirus E4 promoter [34] where more than 5–16-fold larger calculated ΔA_np_ over the observed ΔA_np_ was reported. Small deviations in the calculated ΔA_np_ over the observed ΔA_np_ (within 2-fold) are thought to be insignificant, and thus represent a rigid body interaction [35]. For the Herceptin interaction, the calculated and the observed buried ΔA_np_ in the crystal structure match closely (ΔΔA_np_ = 30 Å^2^, 3% of the observed ΔA_np_), indicating minimal effect of the molecular rearrangements upon binding, consistent with the small differences between the free and bound structures of Herceptin and HER2 [23], [24] (**Figure S2**). For bH1-44, there is also reasonable agreement with the calculated buried area slightly larger than that observed in the crystal structure of the bH1 complexes (by 14% and 18%, or ΔΔA_np_ = 130 Å^2^ and 180 Å^2^ for VEGF and HER2, respectively). The small deviations could be due to errors in the determination of the apolar interfaces based on the crystal structures or the mutations that distinguish bH1 from bH1-44, and indicate that the effect of structural rearrangement on the binding of bH1-44 to either antigen is negligible. Although a structure of free bH1-44 is not available for calculations of solvent accessible surface area, the thermodynamic data suggest that no significant refolding of the CDR loops involving changes in exposure of hydrophobic groups concurs with antigen binding.

### bH1-44 and Herceptin interactions exhibit similar conformational entropy change

As bH1 exhibits highly distinct conformations in the HER2 and VEGF complexes, it is possible that the CDR loops are rather flexible in the unbound state. We therefore examined the extent of the entropy changes due to the loss of conformational freedom upon bH1-44/HER2 interaction versus Herceptin/HER2 interaction. We dissected the total entropy change observed (ΔS_TOT_), which is comprised of entropy changes from desolvation effects (ΔS_SOLV_), reduction of rotational and translational freedom (ΔS_RT_), and changes in internal conformational freedom (ΔS_CONF_) (ΔS_TOT_ = ΔS_SOLV_+ΔS_RT_+ΔS_CONF_) [36]. Based on the heat capacity change (ΔCp) described above, ΔS_SOLV_ was calculated as 96 cal/molK^−1^ for bH1-44/VEGF, 105 cal/molK^−1^ for bH1-44/HER2 and 89 cal/molK^−1^ for Herceptin/HER2 (**Table 2**). ΔS_RT_ was estimated to contribute −8 cal K^−1^ mol^−1^ for all interactions [36], [37], and we derived ΔS_CONF_ to be −72 cal K^−1^ mol^−1^ for bH1-44/VEGF, −64 cal K^−1^ mol^−1^ for bH1-44/HER2, and −70 cal K^−1^ mol^−1^ for Herceptin/HER2 (**Table 2**). Thus, HER2 binding by Herceptin and bH1-44 involves a similar conformational entropy penalty. The slightly larger entropic penalty from reduction in the conformational freedom for the bH1-44/VEGF interaction relative to the bH1-44/HER2 interaction may reflect what is observed in the crystal structures of free VEGF showing multiple conformations in the regions bound by bH1 [38]. The crystal structures of HER2 did not reveal such structural diversity [22], [24]. Thus, compared to the Herceptin/HER2 interaction, the bH1-44 interactions with HER2 or VEGF are associated with relatively similar extents of entropic penalty due to the reduction of conformational freedom.

### Temperature dependence of association rate constants of bH1-44 and Herceptin interactions is similar

We next examined the temperature dependence of the association rate constants to assess the activation energy for complex association as described in other studies [35], [39], [40], [41]. The activation energy is a measure of the energy required to reach the transition state for association or dissociation. Large activation energy of association has been proposed to result from the energetic costs of ordering a flexible binding site for interaction [40]. The activation energy for dissociation on the other hand is indicative of the degree of complex stabilization.

By examining the binding kinetics of bH1-44 or Herceptin by SPR measurements at temperatures ranging from 5°C to 37°C, we found that the associate rate constants (k_on_) of bH1-44 interaction with HER2 or VEGF displayed a similar temperature dependence as Herceptin Fab interaction with HER2, *i.e.*, the slopes of linear regression of *ln* k_on_ plotted against 1/T were very similar (linear coefficient = 0.973, 0.998, 0.972, respectively) (**Figure S8**) (See Methods). Therefore, the bH1-44 paratope appears similarly accessible for either HER2 or VEGF binding as the Herceptin paratope is for HER2 binding. This is consistent with the conclusion above that bH1-44 and Herceptin interactions are subjected to similar entropic penalties due to reduction of conformational flexibility upon antigen binding. The apparent additional structural plasticity required for the dual interaction of bH1-44 relative to Herceptin appears to pose minimal additional energy barriers.

## Discussion

Herein, we examined the interactions of bH1 antibodies with HER2 and VEGF by mutagenesis, structural, biophysical and thermodynamic studies. Both interactions exhibit properties characteristic of antibody-antigen interactions: high affinity, exquisite specificity contributed by a small number of functional hotspot residues, and the size and properties of the complementary interfaces. Conventional wisdom expects that the significant structural plasticity demanded by this dual specificity would result in a high entropic cost. But that is not what we observed. The bH1 interactions, in contrast to Herceptin interaction, are entropy-driven. Dissecting the favorable total entropy changes revealed that the entropic penalty from the reduction of conformational freedom for bH1-44 upon binding to either antigen is similar to that for Herceptin, which exhibits only minimal structural plasticity. Comparing the calculated and observed apolar surface area buried in the interface indicated that the structural rearrangement of the bH1-44 paratope concurrent with antigen binding impacted the binding energy minimally. Thus the distinct conformations of bH1 antibodies for dual binding appear to be at a similar energetic state. These conformations may be present prior to antigen binding as isomers (preexisting equilibrium mechanism), may appear during the binding interaction through rearrangement (induced fit mechanism), or a combination of the two scenarios may occur. Overall, the large desolvation energy or hydrophobic effect is the common driver for the dual-specific bH1 variants to engage both HER2 and VEGF and plays a more dominant role in dual interaction than in the Herceptin-HER2 interaction. Recently, Boulanger et al. reported that large desolvation energy in the absence of any observable plasticity structurally is the main driver for the recognition of multiple distinct ligands by the cytokine receptor gp130 [42]. Hence, water repulsion indeed endows the structure-insensitive entropic energy that can drive the multi-specific interactions in the absence or in the presence of apparent structural plasticity.

In the immune system, structural plasticity plays an important role in the antigen recognition of both antibodies and T cell receptors (TCR) [43], [44] [45], [46]. While the antigen binding sites of antibodies and TCRs have essentially the same fold, they differ in their antigen binding affinities. TCRs typically have low affinity (K_D_ in the range of 1–100 µM) for antigen, which is a peptide fragment loaded major histocompatibility complex (pMHC), whereas antibodies are often matured to high affinities (in the nanomolar range). The low affinity TCR-pMHC interactions often involve structural plasticity of the antigen binding sites that significantly impacts the binding energetics as manifested by large conformational entropy penalties, significant local folding effects (large deviations between the observed and the calculated buried apolar surface area) and/or large activation energies [33], [40], [41], [46]. Although the structural plasticity of TCRs contributes to low affinity it may play an important role for T cells in distinguishing between MHCs presenting self and non-self peptides, thus regulating the immune response [33], [41]. The low affinity requirement for TCR function may explain why the commonly observed structural plasticity for TCR interactions with their natural ligands rarely associates with positive entropic contribution. In contrast, the mission of antibodies is to capture antigens efficiently. The affinities of antibodies are improved by somatic mutations that can stabilize and fix the free paratope conformations for antigen binding thereby reducing the entropic penalty upon binding [47], [48]. Here, we show that structural plasticity of antibodies can occur without large entropic penalties and allow high affinity antigen binding, suggesting that increasing the structural plasticity (or adaptability) without increasing the entropic cost may be a general mechanism for antibody maturation as well as antibody multi-specificity.

The available structural data have revealed that structural plasticity is a common and important phenomenon at the site of molecular interactions [49], [50]. Structurally accessible and adaptable (plastic) regions of natural proteins often evolve as sites for ligand interaction [51], [52]. Further, structurally plastic regions have been shown to preferentially attract new binding ligands in vitro by repertoire selection of non-biased libraries of peptides and proteins [49], [51]. The adaptability of molecular surfaces appears to play a key role for the capacity for interaction. Our comprehensive analysis of the high affinity bH1 antibodies reveals how antibody-antigen interactions characterized by entropy-driven binding interactions can occur in the presence of significant conformational plasticity. The combination of these biophysical and structural properties are likely important not only for bH1, but also for other antibodies to evolve binding specificity to one or more antigens and contribute to the vast antibody-antigen recognition capacity of the antibody repertoire.

## Methods

### Protein expression, purification and binding analysis

bH1, bH1-81 and bH1-44 antibodies were isolated from phage displayed antibody libraries and cloned into Fab and IgG expression vectors, expressed in *E. Coli* and 293 cells respectively, and purified by protein A affinity chromatography as described [22]. VEGF_8–109_ denotes the amino terminus 8–109 amino acids of the receptor-binding domain of VEGF and was expressed in *E. Coli*, purified and refolded as described [53]. The extracellular domain (ECD) of HER2 was expressed and purified as described [54]. All proteins were finally dialyzed into phosphate buffer saline (PBS) or other buffer as specified, and quantified by UV absorption for characterization.

### Affinity measurements and kinetic analysis

To determine the binding kinetics and affinity we performed an SPR-based assay on a BIAcore 3000. We immobilized VEGF_8–109_ and HER2 ECD on CM5 chips at a density that allowed us to achieve Rmax in the range of 50–150 Response Units (RU). Serial dilutions of Fab in PBS with 0.05% Tween20 were injected at 30 µl/min. The binding responses were corrected for buffer effects by subtracting responses from a blank flow cell. A 1∶1 Langmuir fitting model was used to estimate the k_on_ (on-rate) and k_off_ (off-rate). The K_D_ values were determined from the ratios of k_on_ and k_off_.

### Temperature dependence of binding kinetics

The binding kinetics was measured at a temperature range of 5°C to 37°C using SPR on a BIAcore3000. The natural logarithm of the k_on_ or k_off_ was plotted against the inverse of temperature. The slopes of the line by linear regression were determined. Based on Arrhenius analysis [55], the energy barrier, or activation energy (E_a_) can be derived from the slopes (E_a_ = −slope multiplied by R, R = gas constant). The off-rates for high affinity Herceptin and bH1-44 at low temperatures were not reliable based on standard SPR determination, therefore we do not discuss the energy barrier of dissociation.

### Isothermal titration calorimetry

Microcalorimetric measurements of the interaction between Fabs and human VEGF_8–109_ and HER2 ECD were performed on a VP-ITC titration calorimeter (Microcal Inc.) as described [26]. Protein solutions were extensively dialyzed into phosphate-buffered saline pH 7.4 or 10 mM Tris-HCl pH 7.5. The antigen and Fabs were dialyzed in the same buffer vessel to minimize mixing heat effects due to differences in buffer composition. Fabs at a concentration of 100–220 µM were titrated into antigen solutions (HER2 ECD or VEGF_109_) at a concentration of 10–22 µM. This concentration of antigen was required for precise enthalpy measurements, but precludes determination of the K_D_ in cases where the binding affinity was high. 15 or 20 injections were performed to obtain a 2-fold excess of antibody. The heats of reaction were determined, heats of Fab dilution were subtracted, and the ΔH was calculated. The bH1 was the only variant with an affinity sufficiently low to be estimated by ITC. The K_D_ was in good agreement with the values obtained by SPR. For all antibodies the dissociation constants (K_D_) determined by surface plasmon resonance were used to determine the binding free energy (ΔG) according to:
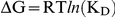
The binding free energy (ΔG) and the enthalpy change (ΔH) determined by ITC allowed the calculation of the change in entropy upon association (ΔS) according to:
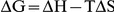
or
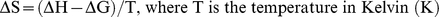
For determination of the heat capacity, ΔCp, microcalorimetric measurements were performed as described above at different temperatures ranging from 20°C to 37°C (293–310°K). The ΔCp was determined by linear regression by plotting ΔH as a function of the temperature.

### Differential scanning calorimetry

Thermal denaturation experiments were performed on a differential scanning calorimeter from Microcal Inc. Fabs were dialyzed against 10 mM sodium acetate pH 5, 150 mM sodium chloride. The solutions were adjusted to a concentration of 0.5 mg/ml and heated to 95°C at a rate of 1°C/min. The melting profiles were baseline corrected and normalized. The T_M_ was determined using the software supplied by the manufacturer.

### Characterization of the bH1/VEGF and bH1/HER2 structural interface

The biochemical composition of the binding interface was calculated using the SOLV function in the program XSAE (from Dr. C. Broger, Hoffmann-La Roche, Basel, Switzerland). This program calculates the surfaces of selected binding fragments/chains in structures. The area was calculated using a probe with a radius of 1.4 Å. For each atom the solvent accessible surface area and the area occluded by atoms of the other chain (both in Å^2^) are calculated. The program designates the occlusions as polar, hydrophobic or mixed. “Mixed” means occlusion of polar atoms on one chain by hydrophobic atoms on another chain. We estimated the buried hydrophobic surface area as the purely hydrophobic occlusions plus half of the mixed. All structural figures were generated using Pymol (by W. DeLano).

### Construction of bH1-44 and Herceptin mutants

A vector that encoded the Fab fused to the N-terminus of geneIII via the heavy chain was used as the template for Kunkel mutagenesis [56]. Oligonucleotides were designed to introduce the desired alanine mutations at selected positions. The Fab Ala mutants were expressed as phage and the relative binding affinity estimated by competition ELISA. The heavy chain and the light chain variable domains were then cloned into Fab and IgG expression vectors, and Fabs and IgGs expressed and purified as described [22]. SDS PAGE verified the correct protein size and size exclusion chromatography confirmed aggregation levels below 5%.

### Dissection of the entropy change

The total entropy change (ΔS_TOT_) can be divided into contributions from desolvation effects (ΔS_SOLV_), entropy changes from the loss of rotational and translational degree of freedom (ΔS_RT_) and changes in configurational and conformational flexibility and dynamics of the interacting molecules (ΔS_CONF_) [36]. This can be expressed as:
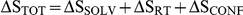
Typically, only ΔS_SOLV_ is positive while ΔS_RT_ and ΔS_CONF_ are both negative. ΔS_RT_ is estimated to contribute −8 cal K^−1^ mol^−1^ (the cratic entropy term) for the association of two molecules as described [36], [37]. ΔS_SOLV_ is dominated by the hydrophobic effect due to the burial of apolar surface area and can be described as a function of ΔCp:
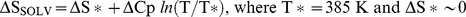
ΔS_CONF_ can thus be estimated as:
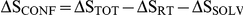



## Supporting Information

Movie S1 **Movie modeling the molecular acrobatics of CDRs for bH1 dual interaction.** The significant structural plasticity of bH1 is highlighted by morphing between VEGF-bound bH1 (PDB code 3BDY) and HER2-bound bH1 (PDB code 3BE1) presented as described in Figure S1. Please note the highly distinct conformation of CDR-L1, the adjustment of the side chains of highlighted residues, and the side chains of LC-I30c and LC-Y32 that alternatively occupy the nearby cavity in HER2 bound bH1 or VEGF bound bH1, respectively. The movie (see movie S1) has been generated using the morphing function of Rigimol which is part of MacPymol 1.3 (www.pymol.org).
(MOV)

Figure S1 **The structural plasticity of bH1 CDRs.** The three HC (in blue) and three LC CDRs (in yellow) of bH1 are shown in cartoon representation with the rest of bH1 structure as surface of the VEGF bound form. The side chains of selected residues as in Figure 1 are shown in stick representation. Please see Movie S1 for the extent of bH1 CDR movements for its dual binding mode.
(TIF)

Figure S2 **Superimposition of Herceptin as Fv and Fab in their unbound form with HER2-bound Fab shows small conformational variability.** The CDRs of representative protamers of the Herceptin variable fragment (Fv) (orange, PDB entry 1FVC) and of the Herceptin Fab (olive and teal, PDB entry 1FVD) – both in the unbound form – are shown in cartoon representation. Both structures were superimposed with the Herceptin Fab bound to the ECD of HER2 (blue, PDB entry 1N8Z). Three different conformations of the CDR-H3 region in the unbound Herceptin fragments were observed, one of them (in teal) being similar to the CDR-H3 conformation observed in the HER2 bound Fab. In the unbound structure, CDR-H3 with residue Y105 (or Y100a as Kabat numbering) shown in stick representation is involved in crystal packing explaining the different conformation of the CDR-H3 loop (Eigenbrot, C. et al. J. Mol. Biol. *229*, 969–995, 1993).
(TIF)

Figure S3 **Distinct residues contributing to VEGF- or HER2-binding.** The residues that are important for VEGF binding LC-I29 and LC-Y32 (A and B) and for HER2 binding HC-R50 and HC-R58 (C and D) are shown as cyan sticks on the bH1/VEGF (A and C, 2.6 Å resolution) or bH1/HER2 (B and D, 2.9 Å resolution) crystal structures (Bostrom *et. al.*, Science 323, p1610, 2009). The residues I29 and Y32 appear to be involved in intra-chain interactions that serve to maintain the CDR-L1 loop conformation necessary for VEGF-binding. In the HER2-bH1 structure, I29 is solvent exposed and Y32 packs against HER2 but does not engage in functionally important interaction. R50 and R58 pack against D560 and E558 on HER2 and appear to engage in charge-charge interactions. R50 and R58 are solvent exposed in the VEGF complex structure.
(TIF)

Figure S4 **Herceptin Fab binding to HER2 is severely disrupted by the same mutations that abolish bH1-44 interaction with HER2.** (A) The kinetics of the Fabs binding to immobilized HER2 was determined using surface plasmon resonance at 30°C using a 1∶1 Langmuir binding fitting model. Error bar represents the standard deviations (SD) of more than three independent experiments. NB = No binding detected. (B) SDS-PAGE of the R50A, R58A and R50A/R58A Herceptin mutant Fabs (non-reduced). The expressed two-chain Fabs appear sufficiently folded to associate as covalently linked heterodimer.
(TIF)

Figure S5 **Thermostability of the dual specific Fabs.** bH1 Fab (A), bH1-81 Fab (B), and bH1-44 Fab (C) at 0.5 mg/ml were heated to 95°C at a rate of 1°C/min. The melting profiles were baseline corrected and normalized. The black trace represents the measured melting curve and the red trace the fitted curve. The melting temperatures (Tm) were calculated from the fitted curves according to the manufacturer's instructions to 77°C, 76°C, 74°C for bH1, bH1-81 and bH1-44, respectively.
(TIF)

Figure S6 **Representative calorimetric measurements of the enthalpy changes associated with Fab-antigen binding at 30°C.** The individual heat pulses (top) and the heats of reaction (bottom), which are calculated by integration of each pulse, are plotted as a function of the antibody to antigen ratio for each injection. The small magnitude of the enthalpy changes required relatively high protein concentrations, which precluded accurate estimation of the K_D_ when the affinity was high. Solutions of VEGF_109_ or HER2-ECD at concentrations ranging from 10–20 µM were titrated by 15 injections of bH1 or bH1-44 Fab at concentrations from 100 to 200 µM in PBS. (E–F) Solutions of VEGF_109_ or HER2-ECD at concentrations of 10 to 20 µM were titrated by 20 injections of bH1-44 LC-I29A+Y32A Fab or bH1-44 HC-R50A+R58A Fab at concentrations of 150 and 250 µM in PBS. Titrations 1 and 13 in (E) were excluded from the analysis due to instrument noise.
(TIF)

Figure S7 **The heat capacity changes associated with bH1-44 Fab interactions.** ΔCp was determined from the slope of the temperature dependence of ΔH between 20°C and 37°C. In this range, ΔCp appears to be independent of T, based on the linear relationship between ΔH and T (Linear coefficient R = 0.9991 for bH1-44/HER2, R = 0.9989 for bH1-44/VEGF).
(TIF)

Figure S8 **Temperature dependence of the association rate constants (k_on_).** The Arrhenius plots (linear regression of *ln*k_on_ versus 1/T ) for bH1-44 interacting with HER2 (open circle), VEGF (open square) and Herceptin Fab (close circle) interacting with HER2 are shown, with linear correlation coefficient of 0.973, 0.998, 0.972, respectively. The slopes are used to derive the energy barrier or activation energy of association, E_a_^ass^ (E_a_ = −(slope)×R, R = gas constant). E_a_^ass^ in kcal/mol is denoted.
(TIF)
