# Supplementary figures and images for: High Affinity Antigen Recognition of the Dual Specific Variants of Herceptin Is Entropy-Driven in Spite of Structural Plasticity
Source: PLoS One. 2011 Apr 22;6(4):e17887. doi: 10.1371/journal.pone.0017887 (PMC3081289; doi:10.1371/journal.pone.0017887)

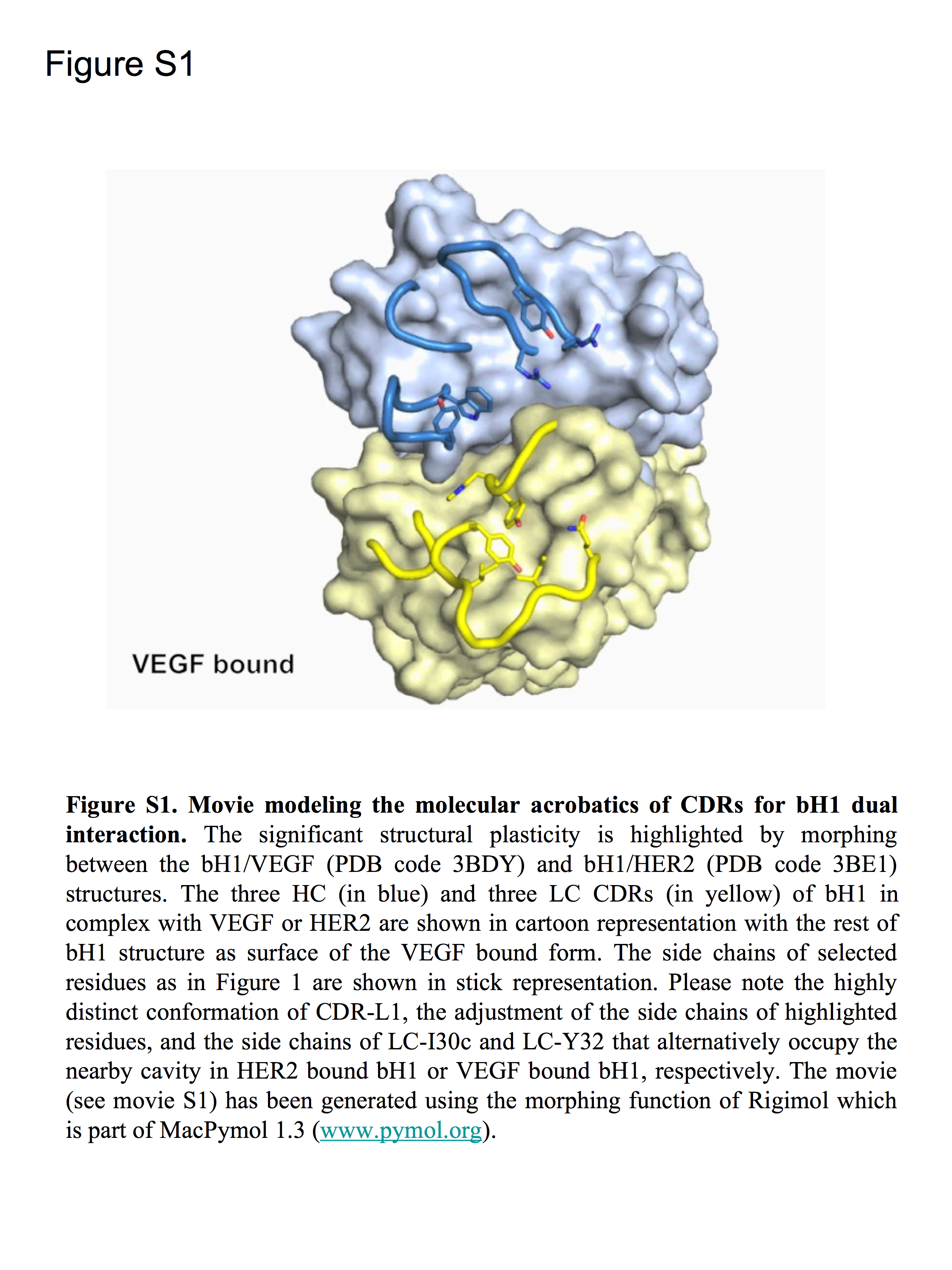

Supplement: Figure S1 — The structural plasticity of bH1 CDRs. The three HC (in blue) and three LC CDRs (in yellow) of bH1 are shown in cartoon representation with the rest of bH1 structure as surface of the VEGF bound form. The side chains of selected residues as in Figure 1 are shown in stick representation. Please see Movie S1 for the extent of bH1 CDR movements for its dual binding mode. (TIF) [file pone.0017887.s002.tif]

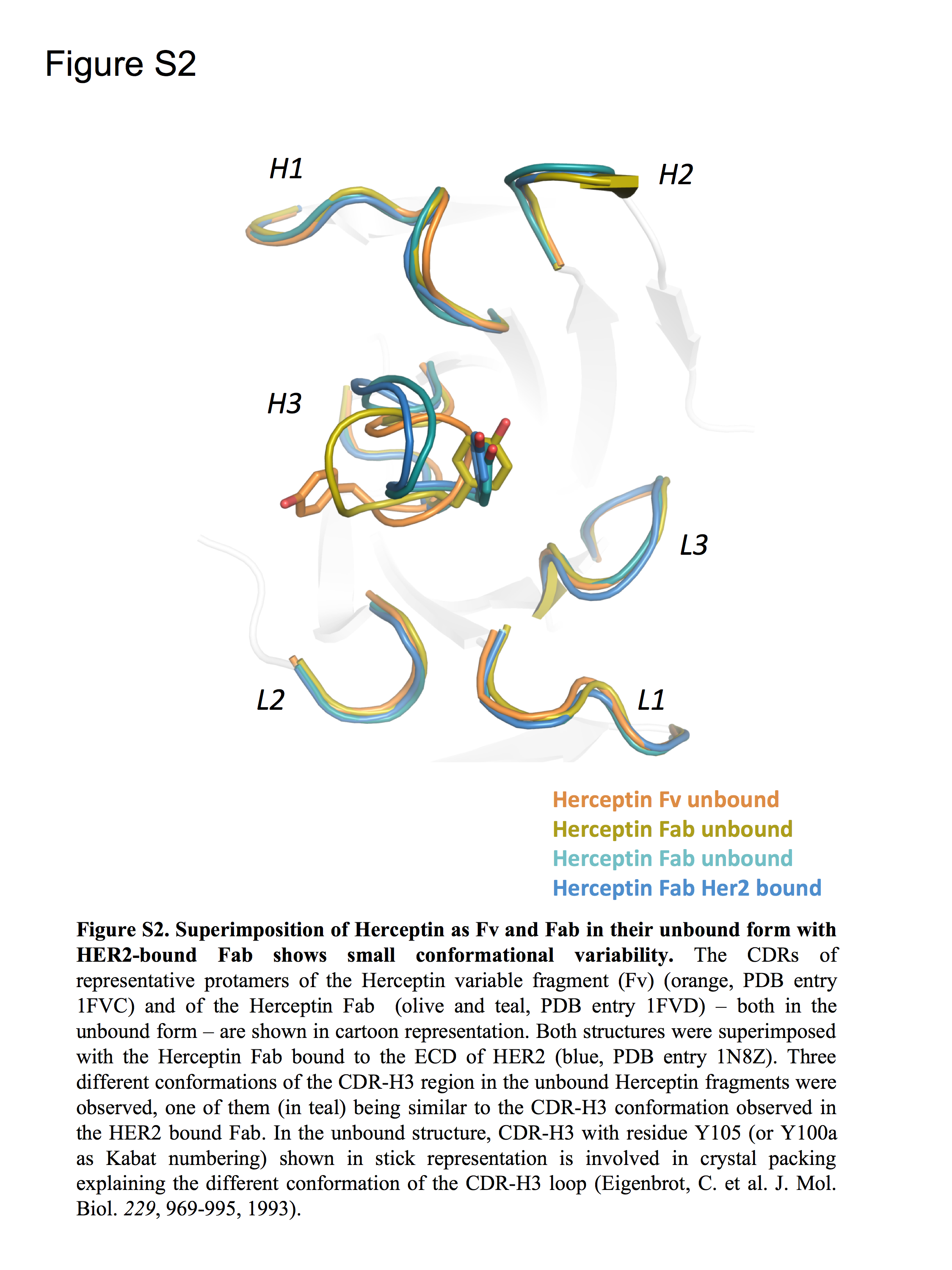

Supplement: Figure S2 — Superimposition of Herceptin as Fv and Fab in their unbound form with HER2-bound Fab shows small conformational variability. The CDRs of representative protamers of the Herceptin variable fragment (Fv) (orange, PDB entry 1FVC) and of the Herceptin Fab (olive and teal, PDB entry 1FVD) – both in the unbound form – are shown in cartoon representation. Both structures were superimposed with the Herceptin Fab bound to the ECD of HER2 (blue, PDB entry 1N8Z). Three different conformations of the CDR-H3 region in the unbound Herceptin fragments were observed, one of them (in teal) being similar to the CDR-H3 conformation observed in the HER2 bound Fab. In the unbound structure, CDR-H3 with residue Y105 (or Y100a as Kabat numbering) shown in stick representation is involved in crystal packing explaining the different conformation of the CDR-H3 loop (Eigenbrot, C. et al. J. Mol. Biol. 229, 969–995, 1993). (TIF) [file pone.0017887.s003.tif]

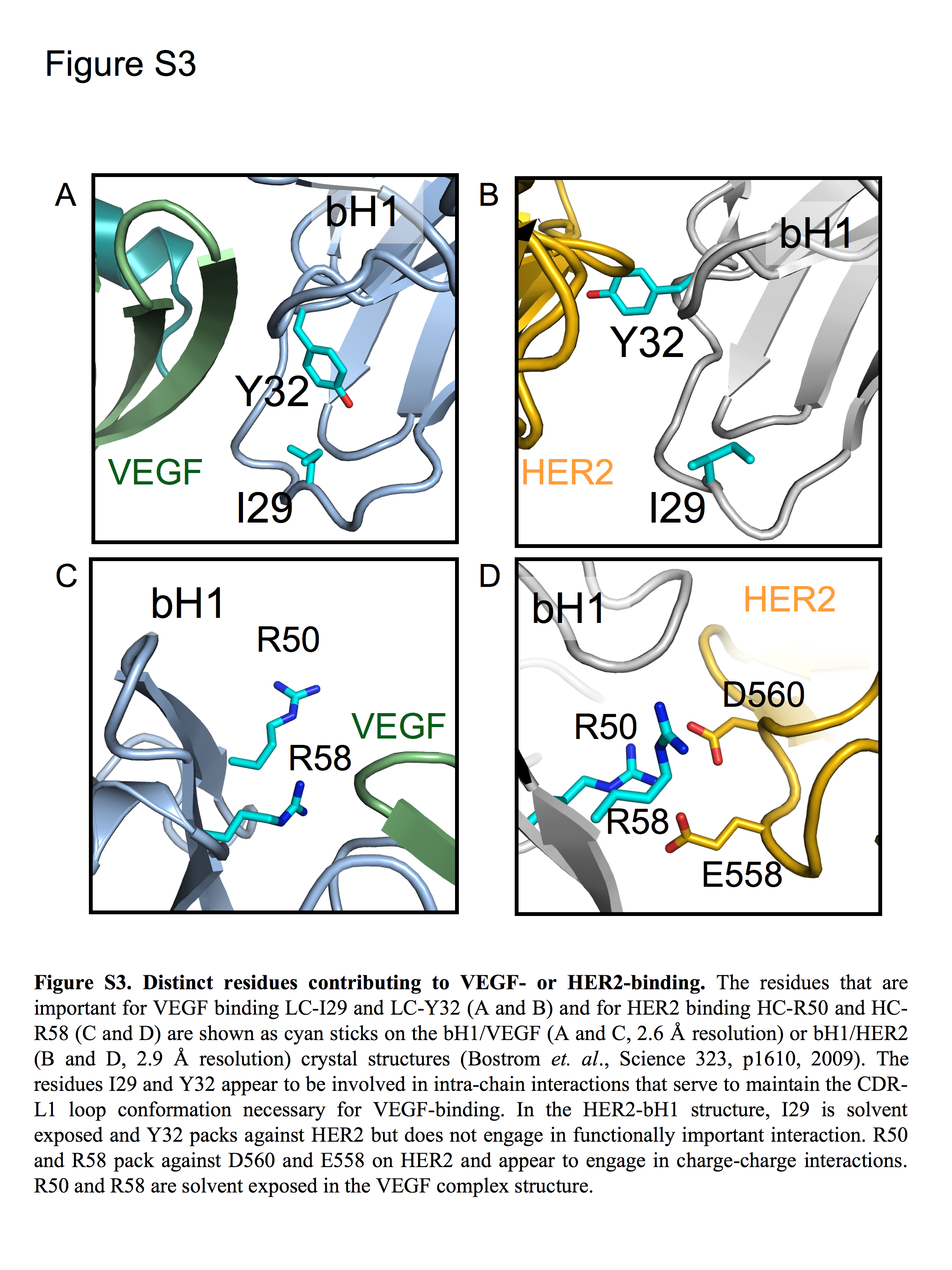

Supplement: Figure S3 — Distinct residues contributing to VEGF- or HER2-binding. The residues that are important for VEGF binding LC-I29 and LC-Y32 (A and B) and for HER2 binding HC-R50 and HC-R58 (C and D) are shown as cyan sticks on the bH1/VEGF (A and C, 2.6 Å resolution) or bH1/HER2 (B and D, 2.9 Å resolution) crystal structures (Bostrom et. al., Science 323, p1610, 2009). The residues I29 and Y32 appear to be involved in intra-chain interactions that serve to maintain the CDR-L1 loop conformation necessary for VEGF-binding. In the HER2-bH1 structure, I29 is solvent exposed and Y32 packs against HER2 but does not engage in functionally important interaction. R50 and R58 pack against D560 and E558 on HER2 and appear to engage in charge-charge interactions. R50 and R58 are solvent exposed in the VEGF complex structure. (TIF) [file pone.0017887.s004.tif]

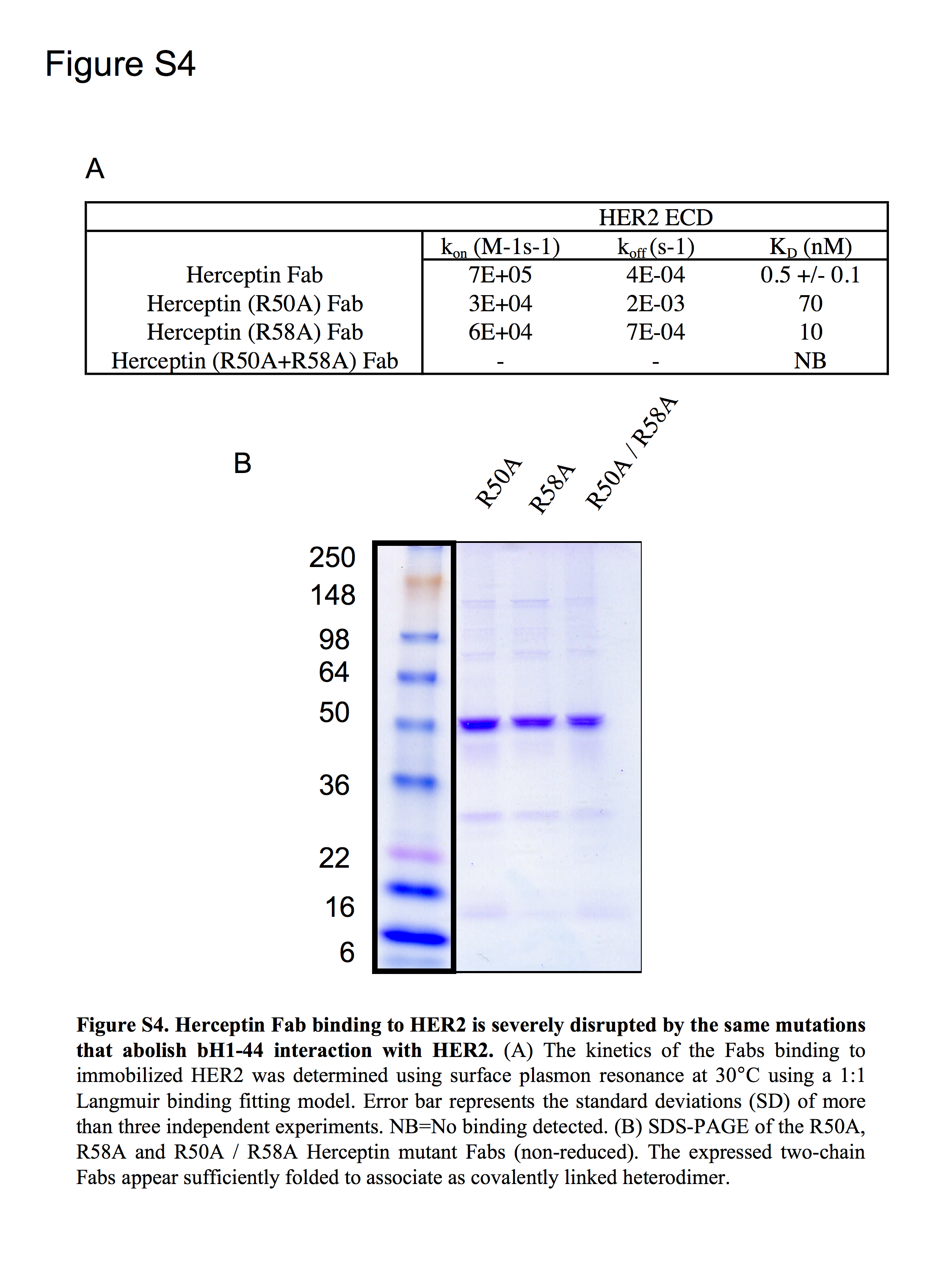

Supplement: Figure S4 — Herceptin Fab binding to HER2 is severely disrupted by the same mutations that abolish bH1-44 interaction with HER2. (A) The kinetics of the Fabs binding to immobilized HER2 was determined using surface plasmon resonance at 30°C using a 1∶1 Langmuir binding fitting model. Error bar represents the standard deviations (SD) of more than three independent experiments. NB = No binding detected. (B) SDS-PAGE of the R50A, R58A and R50A/R58A Herceptin mutant Fabs (non-reduced). The expressed two-chain Fabs appear sufficiently folded to associate as covalently linked heterodimer. (TIF) [file pone.0017887.s005.tif]

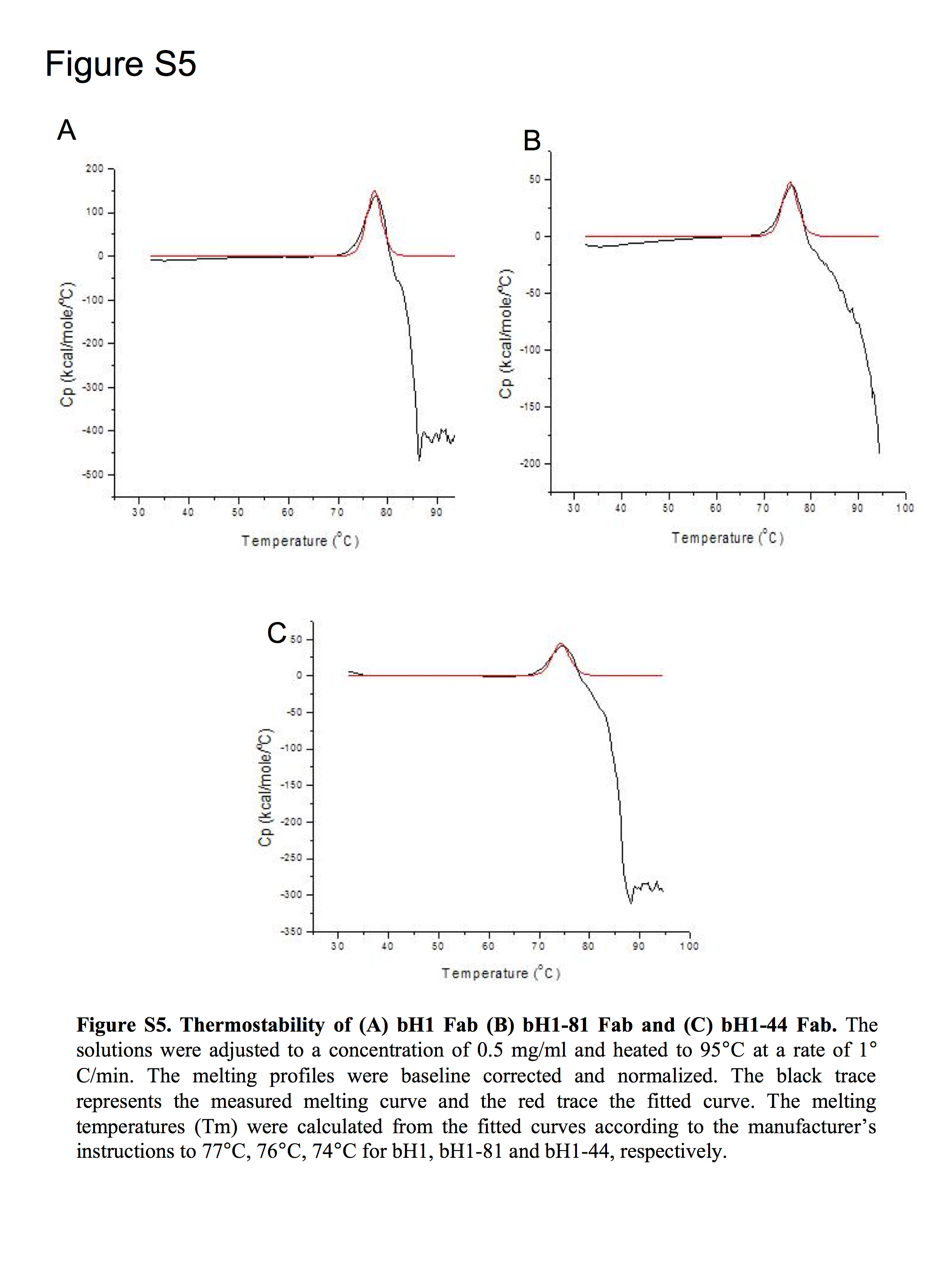

Supplement: Figure S5 — Thermostability of the dual specific Fabs. bH1 Fab (A), bH1-81 Fab (B), and bH1-44 Fab (C) at 0.5 mg/ml were heated to 95°C at a rate of 1°C/min. The melting profiles were baseline corrected and normalized. The black trace represents the measured melting curve and the red trace the fitted curve. The melting temperatures (Tm) were calculated from the fitted curves according to the manufacturer's instructions to 77°C, 76°C, 74°C for bH1, bH1-81 and bH1-44, respectively. (TIF) [file pone.0017887.s006.tif]

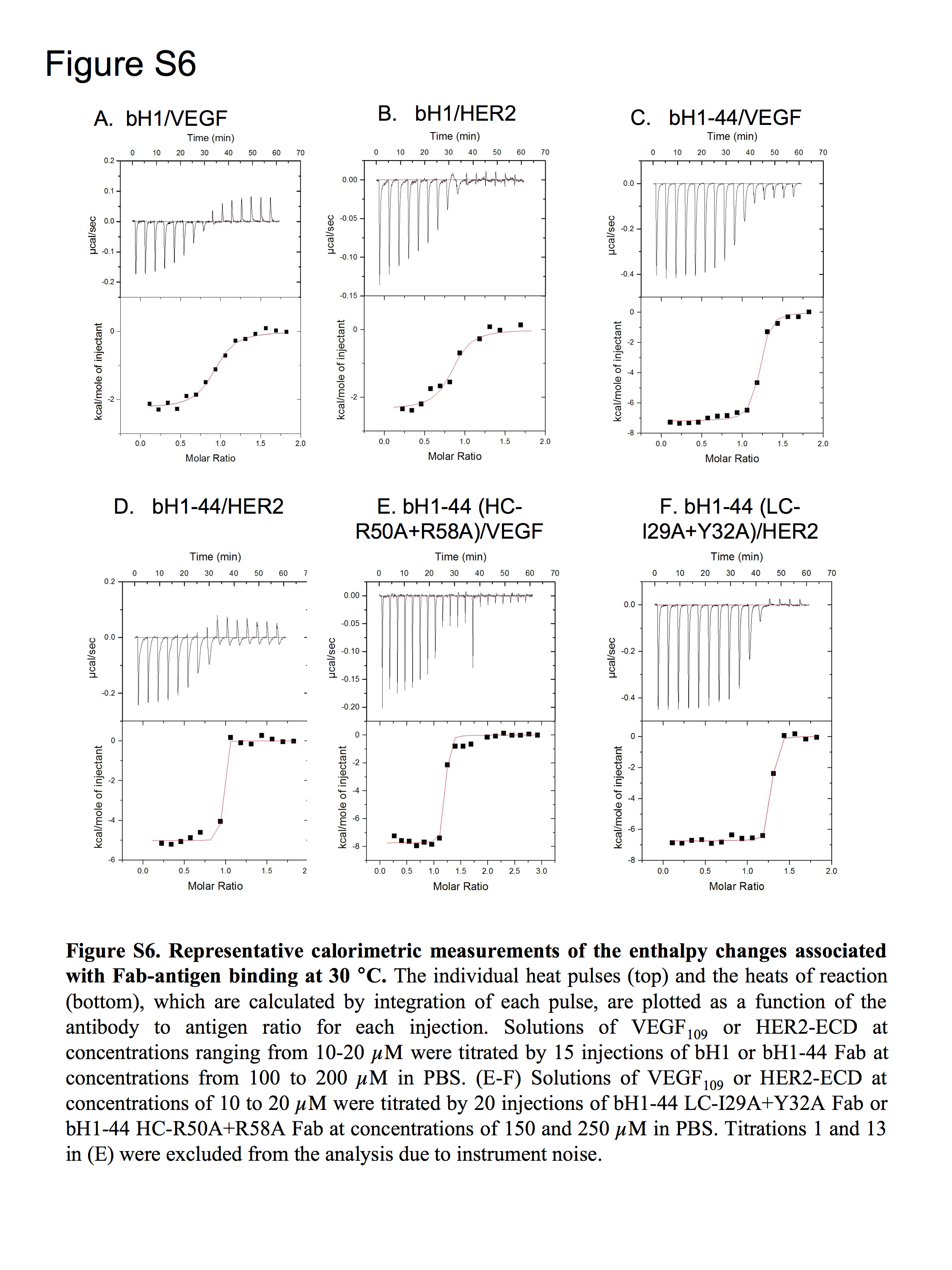

Supplement: Figure S6 — Representative calorimetric measurements of the enthalpy changes associated with Fab-antigen binding at 30°C. The individual heat pulses (top) and the heats of reaction (bottom), which are calculated by integration of each pulse, are plotted as a function of the antibody to antigen ratio for each injection. The small magnitude of the enthalpy changes required relatively high protein concentrations, which precluded accurate estimation of the KD when the affinity was high. Solutions of VEGF109 or HER2-ECD at concentrations ranging from 10–20 µM were titrated by 15 injections of bH1 or bH1-44 Fab at concentrations from 100 to 200 µM in PBS. (E–F) Solutions of VEGF109 or HER2-ECD at concentrations of 10 to 20 µM were titrated by 20 injections of bH1-44 LC-I29A+Y32A Fab or bH1-44 HC-R50A+R58A Fab at concentrations of 150 and 250 µM in PBS. Titrations 1 and 13 in (E) were excluded from the analysis due to instrument noise. (TIF) [file pone.0017887.s007.tif]

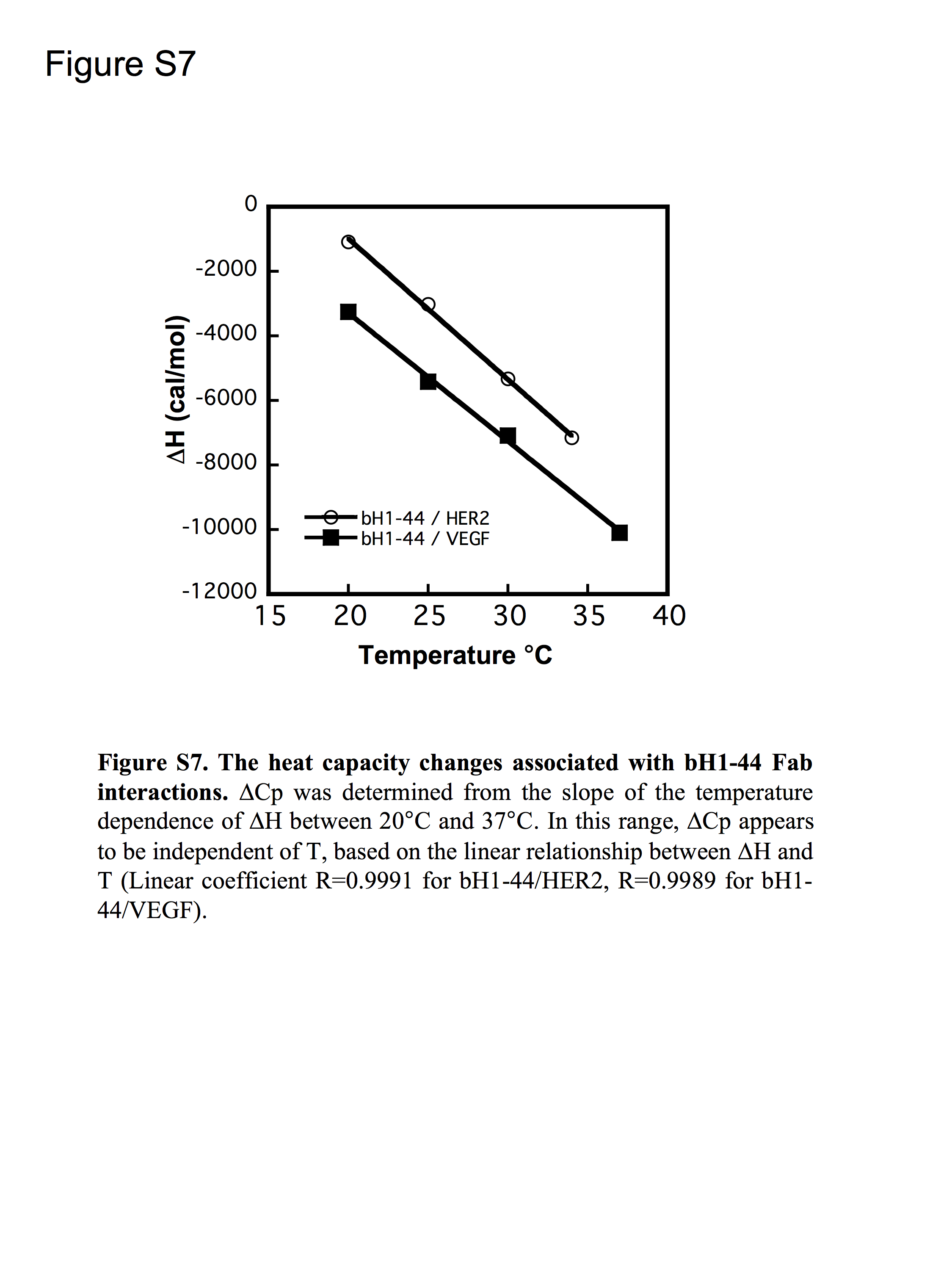

Supplement: Figure S7 — The heat capacity changes associated with bH1-44 Fab interactions. ΔCp was determined from the slope of the temperature dependence of ΔH between 20°C and 37°C. In this range, ΔCp appears to be independent of T, based on the linear relationship between ΔH and T (Linear coefficient R = 0.9991 for bH1-44/HER2, R = 0.9989 for bH1-44/VEGF). (TIF) [file pone.0017887.s008.tif]

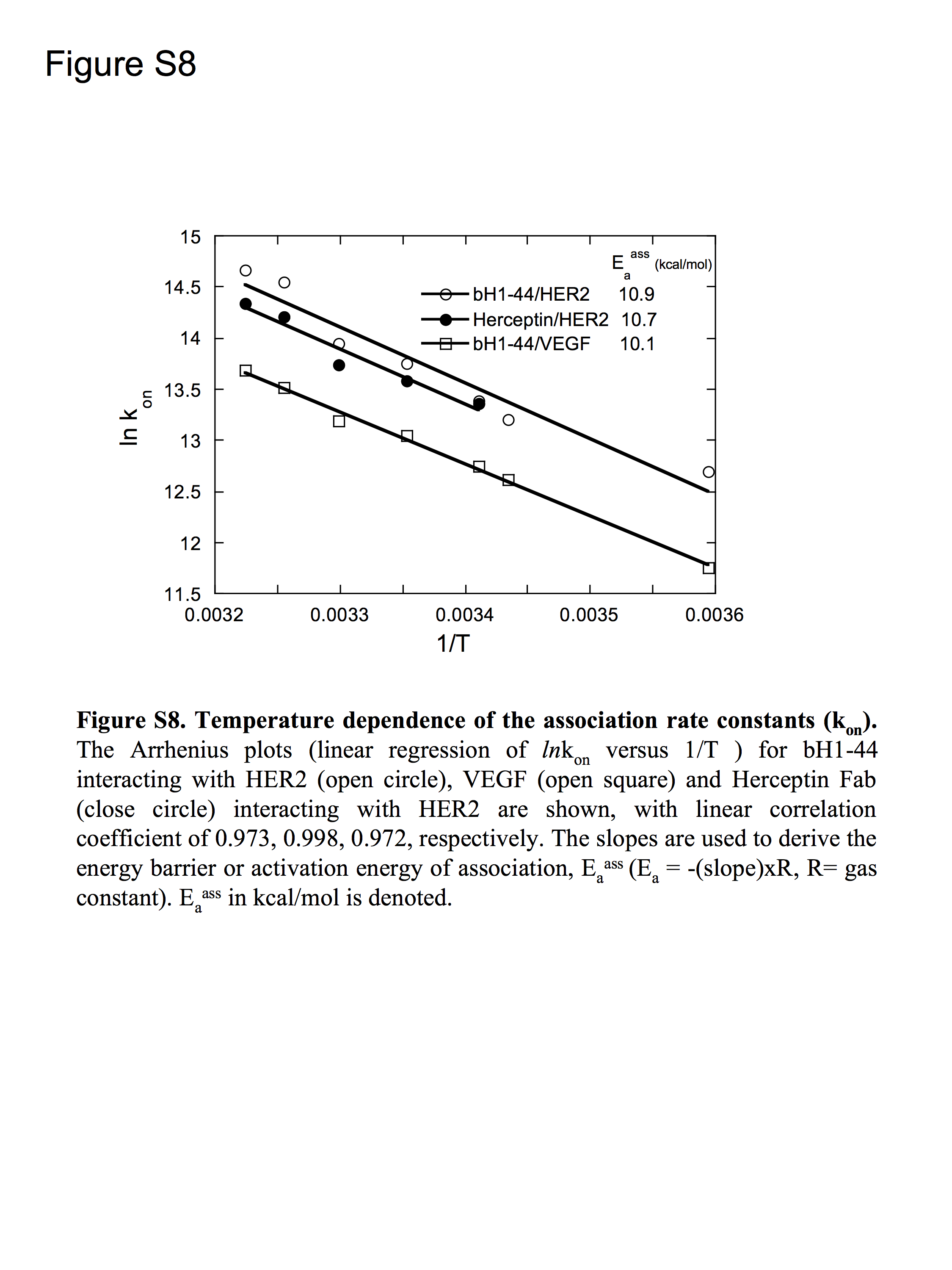

Supplement: Figure S8 — Temperature dependence of the association rate constants (kon). The Arrhenius plots (linear regression of lnkon versus 1/T ) for bH1-44 interacting with HER2 (open circle), VEGF (open square) and Herceptin Fab (close circle) interacting with HER2 are shown, with linear correlation coefficient of 0.973, 0.998, 0.972, respectively. The slopes are used to derive the energy barrier or activation energy of association, Ea ass (Ea = −(slope)×R, R = gas constant). Ea ass in kcal/mol is denoted. (TIF) [file pone.0017887.s009.tif]
